# Association between different metabolic obesity phenotypes and hyperuricemia: the modifying role of liver enzymes

**DOI:** 10.3389/fpubh.2026.1764219

**Published:** 2026-02-20

**Authors:** Ziwei Guo, Aihong Yang, Haobiao Liu, Xuefeng Yu, Jiaxin Liu, Xiang Xiao, Haiyan Li, Jing Han, Zhiyong Du

**Affiliations:** 1 Xi'an Gem Flower Chang Qing Hospital, Xi'an, Shaanxi, China; 2 School of Public Health, Health Science Center, Xi'an Jiaotong University, Xi'an, Shaanxi, China

**Keywords:** hyperuricemia, insulin resistance, liver enzymes, metabolic phenotype, metabolically unhealthy obesity phenotypes, oilfield workers

## Abstract

**Background:**

Hyperuricemia (HUA) is a rapidly increasing metabolic disorder that is closely linked to obesity, nonalcoholic fatty liver disease and cardiometabolic diseases. However, the relationships between different metabolic obesity phenotypes and HUA, and the potential modifying role of liver enzymes, remain unclear, particularly in occupational populations.

**Methods:**

In this cross-sectional study, we enrolled 1,867 Chinese oilfield workers undergoing routine occupational health examinations. Logistic regression models were used to estimate odds ratios (ORs) and 95% confidence intervals (CIs) for HUA. Generalized additive models with penalized smoothing splines were applied to explore potential non-linear associations of BMI and WC with HUA. Stratified analyses were conducted according to liver enzyme status.

**Results:**

The prevalence of HUA was 30.0% among oilfield workers. Both GO and AO were significantly associated with higher odds of HUA after multivariable adjustment (adjusted OR for GO: 1.83, 95% CI: 1.35–2.49; for AO: 1.38, 95% CI: 1.03–1.84). Compared with metabolically healthy non-obesity phenotypes, metabolically unhealthy obesity phenotypes showed the strongest associations with HUA, particularly MUGO (adjusted OR: 4.00, 95% CI: 2.69–6.06) and MUAO (adjusted OR: 3.73, 95% CI: 2.53–5.59). Generalized additive models revealed a linear positive relationship between BMI and HUA risk, whereas the association between WC and HUA was non-linear. Stronger associations between MUGO/MUAO and HUA were observed among individuals with elevated liver enzyme levels.

**Conclusion:**

In this population of oilfield workers, metabolically unhealthy obesity phenotypes, especially MUGO and MUAO, are strongly associated with HUA, with liver enzyme abnormalities acting as potential effect modifiers rather than causal determinants. Metabolic status and liver function appear to be important modifiers of obesity-related HUA risk. Integrating adiposity measures, metabolic components and liver enzyme indices may improve risk stratification and inform targeted prevention strategies for HUA in occupational settings.

## 1 Introduction

Hyperuricemia (HUA) has emerged as one of the fastest-growing metabolic disorders worldwide in recent years, with a prevalence exceeding 14% among Chinese adults and showing a continuous upward trend (1, 2). As a direct manifestation of purine metabolism disorders, HUA not only serves as the major pathological basis of gout but is also closely associated with insulin resistance, metabolic syndrome, NAFLD, and cardiovascular diseases (3–5). Increasing evidence suggests that elevated serum uric acid (SUA) levels are not merely correlated with metabolic abnormalities but may also be involved in their progression, although the directionality of these associations remains incompletely understood (6). Therefore, identifying and understanding the key determinants of HUA are of great significance for the early prevention and control of metabolic diseases.

Obesity is recognized as a major risk factor for HUA; however, the metabolic consequences of obesity vary considerably among individuals (7, 8). Some individuals with obesity maintain normal metabolic profiles despite excessive body weight and are defined as having metabolically healthy obesity (MHO), whereas others exhibit dyslipidemia, insulin resistance and chronic low-grade inflammation, and are categorized as having metabolically unhealthy obesity phenotypes, such as metabolically unhealthy generalized obesity (MUGO) and metabolically unhealthy abdominal obesity (MUAO) (9). In recent years, researchers have increasingly acknowledged that metabolic phenotypes, rather than BMI alone, may better capture the heterogeneity of obesity-related disease risks (10, 11). Nevertheless, findings regarding the association between different metabolic obesity phenotypes and HUA remain inconsistent (12), and evidence from specific occupational populations is still limited.

The liver is the primary organ responsible for purine metabolism and uric acid production, and its functional state is closely associated with the dynamic balance between uric acid synthesis and excretion (13). Liver enzymes such as alanine aminotransferase (ALT), aspartate aminotransferase (AST), alkaline phosphatase (ALP), and *γ*-glutamyltransferase (GGT) are sensitive biochemical indicators reflecting hepatic metabolic activity and cellular injury (14, 15). Elevated liver enzyme levels not only indicate increased hepatic metabolic burden but are also closely associated with insulin resistance and lipid metabolism disorders (15, 16). Previous studies have suggested that hepatic dysfunction may be associated with altered purine metabolism and uric acid handling, potentially contributing to elevated SUA levels (17). Conversely, accumulating evidence also indicates that HUA itself may exacerbate hepatic oxidative stress and inflammation, suggesting a possible bidirectional relationship between liver enzyme elevation and HUA. However, studies investigating the modifying role of liver enzymes in the relationship between metabolic obesity phenotypes and HUA are still scarce.

Occupational and environmental factors may further influence these metabolic processes. Oilfield workers are often exposed to high physical workload, night-shift schedules, and irregular dietary and lifestyle patterns, all of which are associated with energy metabolism disturbances, fat accumulation, and liver dysfunction, thereby potentially increasing the risk of HUA (18, 19). Although previous studies have suggested a significantly higher prevalence of HUA among oilfield workers compared with the general population (20), the specific metabolic characteristics of this population and the underlying role of liver enzymes remain poorly understood.

Therefore, this study aimed to investigate the association between different metabolic obesity phenotypes and HUA among oilfield workers and to further evaluate the potential effect-modifying role of liver enzymes in this relationship. Given the cross-sectional design, this study focuses on identifying associations rather than inferring causality. By elucidating the interacting mechanisms linking hepatic metabolism, obesity phenotypes, and uric acid regulation, this study seeks to provide scientific evidence for risk stratification and early intervention of metabolic disorders in occupational populations.

## 2 Materials and methods

### 2.1 Study design and participants

This cross-sectional study was conducted between October and December 2023 at a hospital in Xi’an, China. The study population consisted of oilfield workers undergoing routine occupational health examinations. The target population comprised employees of a large oilfield enterprise, most of whom were male laborers aged 30–60 years engaged in physically demanding work. Although detailed demographic data for the entire oilfield workforce were unavailable, the study sample was considered representative in terms of occupational and demographic characteristics.

Eligible participants were those aged ≥18 years, nonpregnant, without severe psychiatric disorders, and who provided written informed consent. Trained investigators administered a standardized face-to-face questionnaire to collect demographic characteristics, lifestyle factors, and medical history. Anthropometric and biochemical data were extracted from standardized physical examination records. Given the cross-sectional design, all data were collected at a single time point, and no temporal or causal relationships were assumed.

A total of 3,107 individuals initially met the inclusion criteria. Participants with missing data on WC (*n* = 949) or BMI (*n* = 73), or with incomplete covariate information (*n* = 218), were excluded. Ultimately, 1,867 participants were included in the final analysis, yielding an effective inclusion rate of approximately 60.0%. To assess potential selection bias, baseline characteristics were compared between initially eligible and finally included participants.

The required sample size for this cross-sectional study was estimated using the Equation 1:


(1) $$N=\frac{Z_{1-\alpha /2}^{2}\mathrm{pq}}{d^{2}}$$

Where Z = 1.96 (for *α* = 0.05), *p* = 0.283 [the expected prevalence of metabolic syndrome based on previous studies (21)], *q* = 1−*p*, and the allowable error *d* = 0.1 × *p*. The minimum required sample size was 973. Considering a 20% nonresponse rate, the final required sample size was 1,217. The actual analytic sample of 1,867 participants exceeded this threshold, ensuring sufficient statistical power.

### 2.2 Data measurement

Physical and biochemical measurements were conducted by trained technicians. WC was measured at the level of the umbilicus using a non-elastic measuring tape. Blood pressure (BP) was measured on the right upper arm using an automated sphygmomanometer (Omron HEM-7430) after participants had rested for at least 10 min. Systolic BP (SBP) and diastolic BP (DBP) were recorded.

All participants were required to fast overnight for at least 8 h before venous blood samples were collected the following morning. Biochemical analyses included fasting blood glucose (FBG), triglycerides (TG), high-density lipoprotein cholesterol (HDL-C), SUA, and liver enzymes (ALT, AST, ALP, and GGT).

### 2.3 Assessment of obesity phenotypes

#### 2.3.1 Generalized and abdominal obesity

According to World Health Organization criteria (22), BMI < 18.5 kg/m^2^ was classified as underweight, 18.5–24.9 kg/m^2^ as normal weight, 25.0–29.9 kg/m^2^ as overweight, and ≥30.0 kg/m^2^ as obesity. Given that only 63 participants (3.4%) were underweight, this group was combined with the normal-weight group. Overweight and obese participants were merged into a single “generalized obesity (GO)” group.

Abdominal obesity (AO) was defined following the International Diabetes Federation (IDF) global consensus (23): WC ≥ 90 cm in men or ≥80 cm in women; otherwise, WC < 90 cm (men) or <80 cm (women) was considered non-abdominal obesity.

#### 2.3.2 Definition of metabolic health

Metabolic health was defined according to the 2023 joint interim statement of the IDF Epidemiology and Prevention Task Force (24). Participants were classified as metabolically healthy if none of the following criteria were met: (1) elevated BP: SBP ≥ 130 mmHg or DBP ≥ 85 mmHg, or self-reported history of hypertension or use of antihypertensive medication; (2) elevated TG: ≥1.7 mmol/L; (3) reduced HDL-C: <1.03 mmol/L in men or <1.29 mmol/L in women; (4) elevated FBG: ≥5.6 mmol/L. Participants meeting any one or more of the above criteria were classified as metabolically unhealthy.

#### 2.3.3 Classification of metabolic obesity phenotypes

Based on BMI and metabolic health status, participants were categorized into four metabolic obesity phenotypes: Metabolically healthy non-generalized obesity (MHNGO); metabolically unhealthy non-generalized obesity (MUNGO); metabolically healthy generalized obesity (MHGO); MUGO.

Similarly, according to WC and metabolic health status, participants were classified as: metabolically healthy non-abdominal obesity (MHNAO); metabolically unhealthy non-abdominal obesity (MUNAO); metabolically healthy abdominal obesity (MHAO); MUAO.

### 2.4 Definition of hyperuricemia

According to established domestic and international standards (25), HUA was defined as SUA > 416.4 μmol/L (7.0 mg/dL) in men or >356.9 μmol/L (6.0 mg/dL) in women.

### 2.5 Definition of abnormal liver enzymes

Abnormal liver enzymes were defined as values exceeding the following sex-specific upper limits (26): ALT > 45 U/L (men) or > 34 U/L (women); AST > 35 U/L (men) or > 31 U/L (women); GGT > 38 U/L (men) or > 32 U/L (women); ALP > 128 U/L (men) or > 98 U/L (women). Participants were classified as having elevated liver enzymes if any of the above indicators exceeded the corresponding upper limit; otherwise, they were classified as having normal liver enzymes. This composite binary definition was adopted to reflect overall hepatic dysfunction and to reduce model complexity and potential multicollinearity among highly correlated liver enzyme markers, rather than to attribute effects to a specific enzyme.

### 2.6 Covariates

Covariates included sex, age, marital status, educational level, job type, job tenure, shift work status, dietary habits, taste preference (e.g., light, moderate or salty), habit of eating sweets, smoking status, alcohol consumption, physical activity, and nightly sleep duration. Detailed definitions of covariates are provided in Table 1.

**Table 1:** Distribution of characteristics by hyperuricemia (HUA) status among oilfield workers.

Variables		Total	Non-HUA	HUA	*p*-value
		*n* = 1867	*n* = 1,307	*n* = 560	
Sex, *n*/%	Male	1,235 (66.1)	760 (58.1)	475 (84.8)	<0.001
	Female	632 (33.9)	547 (41.9)	85 (15.2)	
Age (years), *n*/%	23–35	464 (24.9)	280 (21.4)	184 (32.9)	<0.001
	36–50	1,153 (61.8)	842 (64.4)	311 (55.5)	
	51–61	250 (13.4)	185 (14.2)	65 (11.6)	
Marital status, *n*/%	Married	1,561 (83.6)	1,102 (84.3)	459 (82.0)	0.209
	Other (single, divorced, widowed)	306 (16.4)	205 (15.7)	101 (18.0)	
Educational level, *n*/%	Bachelor’s degree or above	542 (29.0)	347 (26.5)	195 (34.8)	<0.001
	Junior college	529 (28.3)	374 (28.6)	155 (27.7)	
	High school or below	796 (42.6)	586 (44.8)	210 (37.5)	
Job type, *n*/%	Oil extraction worker	1,245 (66.7)	873 (66.8)	372 (66.4)	0.878
	Other	622 (33.3)	434 (33.2)	188 (33.6)	
Job tenure, *n*/%	≤13	539 (28.9)	338 (25.9)	201 (35.9)	<0.001
	14–17	425 (22.8)	296 (22.6)	129 (23.0)	
	18–27	520 (27.9)	398 (30.5)	122 (21.8)	
	≥27	383 (20.5)	275 (21.0)	108 (19.3)	
Shift work status, *n*/%	None	289 (15.5)	194 (14.8)	95 (17.0)	0.435
	Yes, without night shifts	456 (24.4)	317 (24.3)	139 (24.8)	
	Yes, with night shifts	1,122 (60.1)	796 (60.9)	326 (58.2)	
Eating pattern, *n*/%	Regular	1,502 (80.4)	1,075 (82.2)	427 (76.3)	0.003
	Irregular	365 (19.6)	232 (17.8)	133 (23.8)	
Taste preference, *n*/%	Light	329 (17.6)	250 (19.1)	79 (14.1)	0.025
	Moderate	1,279 (68.5)	884 (67.6)	395 (70.5)	
	Salty	259 (13.9)	173 (13.2)	86 (15.4)	
Habit of eating sweets, *n*/%	No	1,366 (73.2)	940 (71.9)	426 (76.1)	0.064
	Yes	501 (26.8)	367 (28.1)	134 (23.9)	
Smoking status, *n*/%	Non-smoker	1,172 (62.8)	888 (67.9)	284 (50.7)	<0.001
	Smoker	695 (37.2)	419 (32.1)	276 (49.3)	
Alcohol consumption, *n*/%	Non-drinker	1,450 (77.7)	1,052 (80.5)	398 (71.1)	<0.001
	Drinker	417 (22.3)	255 (19.5)	162 (28.9)	
Physical activity, *n*/%	Regular	668 (35.8)	466 (35.7)	202 (36.1)	0.817
	Occasional	1,051 (56.3)	734 (56.2)	317 (56.6)	
	Never	148 (7.9)	107 (8.2)	41 (7.3)	
Nightly sleep duration (hours), *n*/%	7–8	634 (34.0)	450 (34.4)	184 (32.9)	0.377
	5–6	1,025 (54.9)	705 (53.9)	320 (57.1)	
	<5	208 (11.1)	152 (11.6)	56 (10.0)	
GO, *n*/%	Normal weight	1,001 (53.6)	829 (63.4)	172 (30.7)	<0.001
	Obese	866 (46.4)	478 (36.6)	388 (69.3)	
AO, *n*/%	Normal	1,125 (60.3)	895 (68.5)	230 (41.1)	<0.001
	AO	742 (39.7)	412 (31.5)	330 (58.9)	
Age		41.6 ± 8.0	42.3 ± 7.8	40.0 ± 8.0	<0.001
BMI		24.9 ± 4.0	24.0 ± 3.5	27.1 ± 4.1	<0.001
WC		84.4 ± 11.7	81.6 ± 11.0	90.7 ± 10.7	<0.001
SBP		115 (105, 127)	113 (104, 124)	121 (111, 133)	<0.001
DBP		76 (68, 84)	74 (68, 82)	80 (71, 87)	<0.001
TG		1.6 (1.1, 2.4)	1.4 (1.0, 2.1)	2.2 (1.5, 3.2)	<0.001
HDL-C		1.04 (0.89, 1.22)	1.07 (0.92, 1.25)	0.97 (0.84, 1.13)	<0.001
FBG		4.8 (4.5, 5.2)	4.8 (4.5, 5.2)	4.9 (4.6, 5.3)	<0.001
ALT		21 (14, 32)	19 (13, 27)	28 (19, 45)	<0.001
AST		19 (16, 23)	18 (15, 22)	21 (17, 27)	<0.001
ALP		73 (60, 89)	72 (58, 88)	77 (66, 93)	<0.001
GGT		25 (16, 40)	20 (14, 32)	37 (24, 56)	<0.001

### 2.7 Statistical analysis

All statistical analyses were performed using R software (version 4.5.1) and IBM SPSS Statistics (version 26.0). Continuous variables with a normal distribution were expressed as mean ± standard deviation (SD), while skewed variables were expressed as median (interquartile range). Between-group differences were tested using the t test or one-way ANOVA, and the Kruskal–Wallis test was applied for non-normally distributed data. Categorical variables were summarized as frequencies and percentages and compared using the *χ*^2^ test.

Multivariable logistic regression analysis was conducted to examine the associations between metabolic obesity phenotypes and HUA, with odds ratios (ORs) and 95% confidence intervals (CIs) estimated. A series of progressively adjusted models were constructed: Model 1 was the crude model without adjustment; Model 2 was adjusted for basic demographic characteristics (sex, age, marital status, and educational level); Model 3 was further adjusted for occupational factors (job type, job tenure, and shift work); Model 4 additionally included behavioral and lifestyle variables (regularity of diet, taste preference, habit of eating sweets, smoking status, alcohol consumption, regular physical activity, and nightly sleep duration); and Model 5 was further adjusted for liver enzyme levels (ALT, AST, ALP, GGT).

Generalized additive models (GAMs) were applied to explore potential non-linear associations between continuous adiposity indicators (BMI and WC) and the probability of HUA. Penalized thin plate regression splines were used as smoothing functions, with smoothing parameters automatically selected using generalized cross-validation. The effective degrees of freedom (EDF) were estimated from the data, with EDF values greater than 1 indicating evidence of non-linearity. Covariates were included as linear terms within the GAM framework. The GAM analyses were conducted for exploratory and visualization purposes rather than causal inference. All tests were two-sided, and statistical significance was defined as *p* < 0.05.

## 3 Results

### 3.1 Basic characteristics of oilfield workers and prevalence of HUA

Table 1 presents the basic characteristics of oilfield workers according to HUA status. The mean age of the study population was 41.6 years; 66.1% were male, and the majority had an educational level of high school or below (42.6%). Most participants were married (83.6%), the predominant job type was oil extraction worker (66.7%), and 61.8% were aged 36–50 years. In addition, supplementary analyses comparing included participants with the overall eligible population showed no substantial differences in key demographic and metabolic characteristics, indicating that the study sample was broadly representative (Supplementary Table S1).

Compared with non-HUA participants, individuals with HUA were younger and had a higher proportion of males (both *p* < 0.001), lower educational attainment (*p* < 0.001), and a higher proportion of job tenure ≤13 years (*p* < 0.001). When classified by obesity status, the prevalence of GO and AO was significantly higher in the HUA group than in the non-HUA group (both *p* < 0.001).

Participants with HUA also exhibited higher levels of metabolic parameters, including blood pressure and blood lipids, as well as higher liver enzyme levels (all *p* < 0.001). In addition, the proportions of current smokers and alcohol drinkers were significantly higher among participants with HUA than among those without HUA (both *p* < 0.001). No statistically significant differences were observed between the two groups with respect to marital status, job type, shift work, eating pattern, taste preference, physical activity, or nightly sleep duration (all *p* > 0.05).

### 3.2 Correlations of HUA and liver enzymes with metabolic parameters

**Figure 1:**
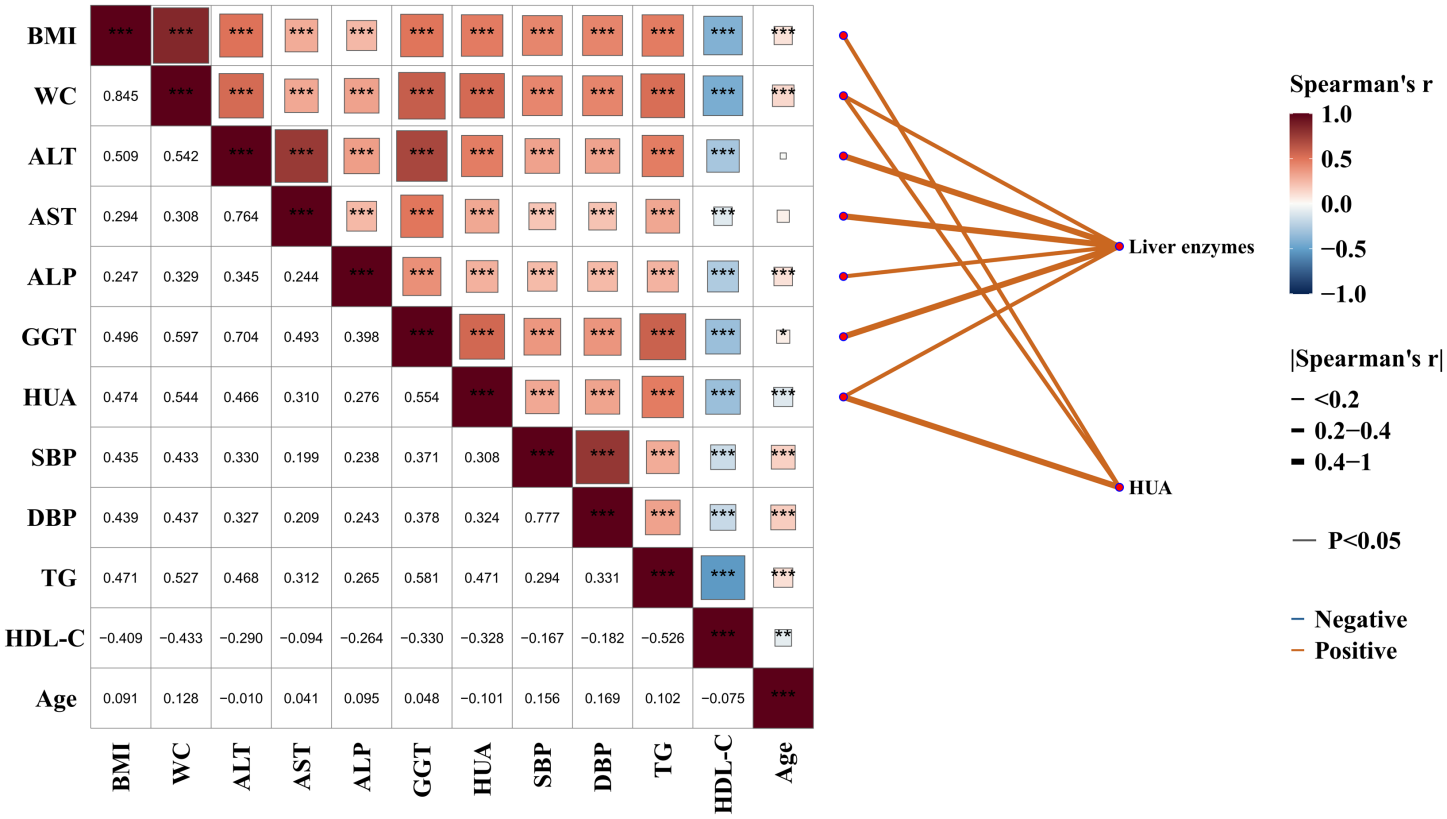
Correlation heatmap and simplified network of associations between HUA, liver enzymes, and metabolic parameters among oilfield workers. The left panel presents Spearman’s rank correlation coefficients (*r*) among BMI, WC, liver enzymes (ALT, AST, ALP, GGT), HUA, blood pressure (SBP, DBP), lipids (TG, HDL-C), and age. Color intensity indicates the strength and direction of correlations (red: positive; blue: negative), and asterisks denote statistically significant correlations (*p* < 0.05). The right panel shows a simplified network diagram illustrating associations of liver enzymes and HUA with metabolic variables. Only statistically significant correlations with an absolute Spearman’s correlation coefficient ≥0.3 are displayed to reduce visual complexity and improve interpretability. Edge color represents correlation direction (orange: positive; blue: negative), and line thickness reflects the magnitude of Spearman’s *r*.

To test our study hypothesis, we used a correlation heatmap network to examine the relationships among HUA, liver enzymes, and various metabolic parameters in oilfield workers (Figure 1). HUA was significantly and positively correlated with BMI, WC, ALT, AST, ALP, GGT, SBP, DBP, and TG (*p* < 0.05). Liver enzymes (ALT, AST, ALP, and GGT) were also significantly and positively correlated with the above obesity- and metabolism-related indices (*p* < 0.05). In contrast, both HUA and liver enzymes were significantly and negatively correlated with HDL-C (*p* < 0.05). These findings indicate that HUA and liver enzymes are significantly associated with multiple metabolic components in this occupational population.

### 3.3 Associations between metabolic obesity phenotypes and HUA

As shown in Table 2, both GO and AO were significantly correlated with an increased risk of HUA. For GO, the presence of generalized obesity was associated with higher odds of HUA in Models 1–5, with ORs of 2.08 (95% CI: 1.56–2.77), 1.83 (95% CI: 1.36–2.46), 1.90 (95% CI: 1.39–2.52), 1.90 (95% CI: 1.41–2.57), and 1.83 (95% CI: 1.35–2.49), respectively, all significantly higher than in participants without GO (*p* < 0.01). For AO, the presence of abdominal obesity similarly correlated with higher odds of HUA, with ORs of 1.42 (95% CI, 1.08–1.86), 1.51 (95% CI, 1.14–2.00), 1.48 (95% CI: 1.11–1.96), 1.48 (95% CI, 1.11–1.96), and 1.38 (95% CI, 1.03–1.84) across Models 1–5.

**Table 2:** Effects of obesity and metabolic components on HUA.

Variables		Model 1	Model 2	Model 3	Model 4	Model 5
GO	No	1.00	1.00	1.00	1.00	1.00
	Yes	2.08 (1.56 ~ 2.77)**	1.83 (1.36 ~ 2.46)**	1.897 (1.39 ~ 2.52)**	1.90 (1.41 ~ 2.57)**	1.83 (1.35 ~ 2.49)**
AO	No	1.00	1.00	1.00	1.00	1.00
	Yes	1.42 (1.08 ~ 1.86)*	1.51 (1.14 ~ 2.00)**	1.48 (1.11 ~ 1.96)*	1.48 (1.11 ~ 1.96)*	1.38 (1.03 ~ 1.84)*
Metabolic components	BP	2.00 (1.36 ~ 2.95)**	1.38 (0.89 ~ 2.15)	1.41 (0.90 ~ 2.20)	1.39 (0.89 ~ 2.18)	1.35 (0.86 ~ 2.12)
	FBG	1.28 (0.81 ~ 2.00)	1.07 (0.64 ~ 1.77)	1.06 (0.64 ~ 1.76)	1.04 (0.62 ~ 1.73)	0.99 (0.59 ~ 1.66)
	TG	3.44 (2.33 ~ 5.10)**	2.24 (1.44 ~ 3.49)**	2.30 (1.48 ~ 3.59)**	2.29 (1.47 ~ 3.58)**	1.98 (1.26 ~ 3.12)**
	HDL-C	0.41 (0.21 ~ 0.81)*	1.14 (0.52 ~ 2.46)	1.08 (0.49 ~ 2.35)	1.05 (0.47 ~ 2.28)	0.81 (0.36 ~ 1.81)

Model 1 is the crude model without any adjustment. Model 2 is additionally adjusted for basic demographic characteristics (sex, age, marital status, educational level). Model 3 further adjusts for occupational factors (job type, job tenure, shift work). Model 4 additionally includes behavioral and lifestyle variables (regularity of diet, taste preference, habit of eating sweets, smoking status, alcohol consumption, regular physical activity, nightly sleep duration). Model 5 is further adjusted, on the basis of Model 4, for liver enzyme levels (ALT, AST, ALP, and GGT). *p* < 0.05*, *p* < 0.01**.

Regarding individual metabolic components, Table 2 shows that elevated TG markedly increased the risk of HUA. For elevated BP, only Model 1 yielded a significantly higher odds of HUA (OR = 2.00, 95% CI: 1.36–2.95; *p* < 0.01) compared with normotensive participants, while no significant associations were observed in Models 2–5. For elevated FBG, the ORs for HUA were not significantly different from those with normal FBG in any model (*p* > 0.05). In contrast, elevated TG was consistently associated with higher odds of HUA, with ORs of 3.44 (95% CI: 2.33–5.10), 2.24 (95% CI: 1.44–3.49), 2.30 (95% CI: 1.48–3.59), 2.29 (95% CI: 1.47–3.58), and 1.98 (95% CI: 1.26–3.12) in Models 1–5, all significantly higher than in participants with normal TG (*p* < 0.01). For low HDL-C, only Model 1 showed a significant association, with an OR of 0.41 (95% CI: 0.21–0.81), indicating a lower odds of HUA compared with those with normal HDL-C (*p* < 0.05), whereas no significant associations were observed in Models 2–5.

In the BMI-based metabolic obesity phenotypes shown in Table 3, using MHNGO as the reference group, the MUNGO was associated with significantly higher odds of HUA in Models 1–5, with ORs of 1.65 (95% CI: 1.13–2.46), 1.78 (95% CI: 1.20–2.69), 1.83 (95% CI: 1.23–2.76), 1.78 (95% CI: 1.20–2.71), and 1.55 (95% CI: 1.04–2.35), respectively (*p* < 0.05 or *p* < 0.01 vs. MHNGO). For the MHGO, only Model 1 yielded a significantly higher odds of HUA (OR = 2.68, 95% CI: 1.37–5.12; *p* < 0.01), while no significant associations were observed in Models 2–5. In contrast, MUGO showed consistently strong associations with HUA across all models, with ORs of 5.94 (95% CI: 4.16–8.69), 5.22 (95% CI: 3.58–7.80), 5.42 (95% CI: 3.70–8.11), 5.36 (95% CI: 3.66–8.04), and 4.00 (95% CI: 2.69–6.06) in Models 1–5, respectively, all significantly higher than MHNGO (*p* < 0.01).

**Table 3:** Effects of different obesity phenotypes on HUA.

Obesity phenotype		Cases (%)	Model 1	Model 2	Model 3	Model 4	Model 5
BMI-based phenotypes	MHNGO	302 (16.2)	1.00	1.00	1.00	1.00	1.00
	MUNGO	699 (37.4)	1.65 (1.13 ~ 2.46)*	1.78 (1.20 ~ 2.69)*	1.83 (1.23 ~ 2.76)**	1.78 (1.20 ~ 2.71)*	1.55 (1.04 ~ 2.35)*
	MHGO	61 (3.3)	2.68 (1.37 ~ 5.12)**	1.91 (0.95 ~ 3.74)	1.89 (0.94 ~ 3.72)	1.94 (0.96 ~ 3.81)	1.78 (0.88 ~ 3.52)
	MUGO	805 (43.1)	5.94 (4.16 ~ 8.69)**	5.22 (3.58 ~ 7.80)**	5.42 (3.70 ~ 8.11)**	5.36 (3.66 ~ 8.04)**	4.00 (2.69 ~ 6.06)**
WC-based phenotypes	MHNAO	314 (16.8)	1.00	1.00	1.00	1.00	1.00
	MUNAO	811 (43.4)	1.95 (1.37 ~ 2.84)**	2.01 (1.39 ~ 2.97)**	2.08 (1.43 ~ 3.08)**	2.04 (1.40 ~ 3.02)**	1.73 (1.19 ~ 2.57)*
	MHAO	49 (2.6)	2.34 (1.12 ~ 4.69)*	1.90 (0.88 ~ 3.94)	1.92 (0.89 ~ 4.00)	1.94 (0.89 ~ 4.03)	1.66 (0.76 ~ 3.46)
	MUAO	693 (37.1)	5.46 (3.86 ~ 7.90)**	5.16 (3.57 ~ 7.62)**	5.28 (3.64 ~ 7.81)**	5.19 (3.58 ~ 7.69)**	3.73 (2.53 ~ 5.59)**

Model 1 is the crude model without any adjustment. Model 2 is additionally adjusted for basic demographic characteristics (sex, age, marital status, educational level). Model 3 further adjusts for occupational factors (job type, job tenure, shift work). Model 4 additionally includes behavioral and lifestyle variables (regularity of diet, taste preference, habit of eating sweets, smoking status, alcohol consumption, regular physical activity, nightly sleep duration). Model 5 is further adjusted, on the basis of Model 4, for liver enzyme levels (ALT, AST, ALP, and GGT). MHNGO, metabolically healthy non-generalized obesity; MUNGO, metabolically unhealthy non-generalized obesity; MHGO, metabolically healthy generalized obesity; MUGO, metabolically unhealthy generalized obesity; MHNAO, metabolically healthy non-abdominal obesity; MUNAO, metabolically unhealthy non-abdominal obesity; MHAO, metabolically healthy abdominal obesity; MUAO, metabolically unhealthy abdominal obesity. *p* < 0.05*, *p* < 0.01**.

In the WC-based metabolic obesity phenotypes in Table 3, using MHNAO as the reference group, MUNAO was associated with significantly increased odds of HUA, with ORs of 1.95 (95% CI: 1.37–2.84), 2.01 (95% CI: 1.39–2.97), 2.08 (95% CI: 1.43–3.08), 2.04 (95% CI: 1.40–3.02), and 1.73 (95% CI: 1.19–2.57) across Models 1–5 (*p* < 0.01 vs. MHNAO). For MHAO, only Model 1 showed a significant association (OR = 2.34, 95% CI: 1.12–4.69; *p* < 0.05), while Models 2–5 yielded no significant associations. MUAO exhibited the strongest and most consistent associations, with ORs of 5.46 (95% CI: 3.86–7.90), 5.16 (95% CI: 3.57–7.62), 5.28 (95% CI: 3.64–7.81), 5.19 (95% CI: 3.58–7.69), and 3.73 (95% CI: 2.53–5.59) in Models 1–5, all significantly higher than MHNAO (*p* < 0.01). These results suggest that metabolic status plays a pivotal role in the relationship between obesity and HUA.

### 3.4 GAM-based smooth curves for the relationships of BMI and WC with HUA

**Figure 2:**
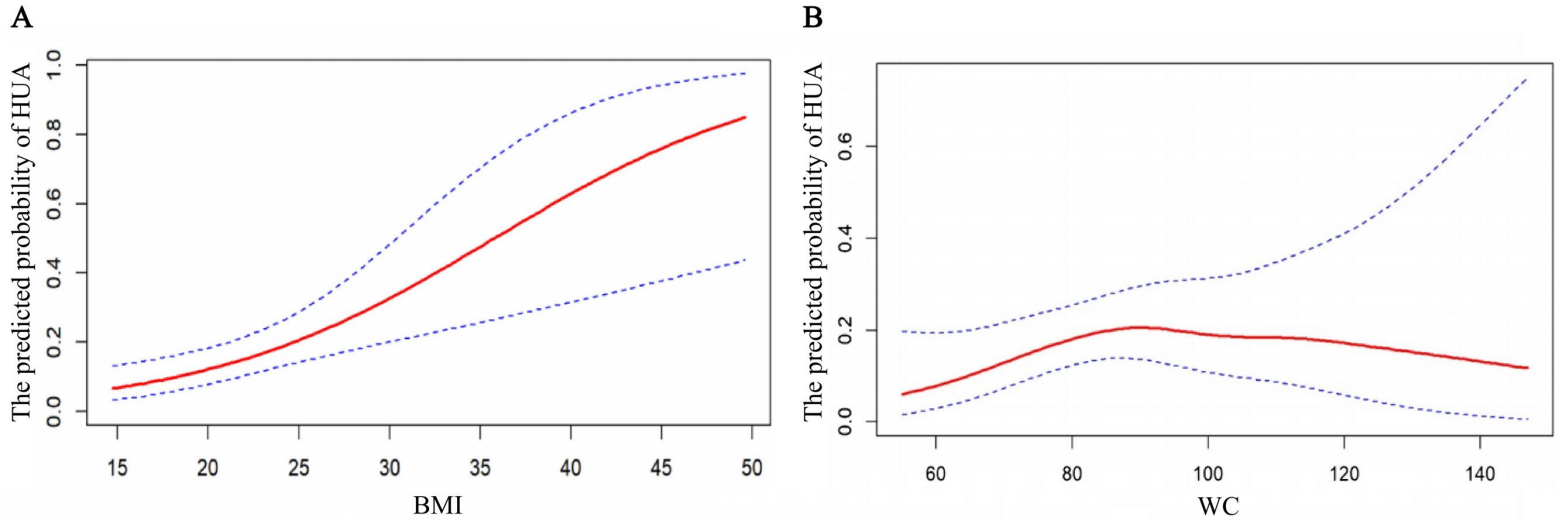
Generalized additive model (GAM)–derived smooth curves illustrating the associations between adiposity measures and the predicted probability of HUA among oilfield workers. **(A)** Association between BMI and the predicted probability of HUA. **(B)** Association between WC and the predicted probability of HUA. The red solid lines represent the fitted probabilities of HUA across the observed ranges of BMI and WC, while the blue dashed lines indicate the corresponding 95% confidence intervals.

To examine the associations between BMI, WC, and HUA, we applied GAMs with smooth curves, as shown in Figure 2. GAMs were fitted using thin plate regression splines, with smoothing parameters selected via generalized cross-validation. Effective degrees of freedom for each spline term were automatically determined. Covariates including age, sex, educational level, marital status, job type, shift work, smoking, and alcohol consumption were included as linear terms in the GAM framework.

As BMI increased, the predicted probability of HUA showed a continuously rising trend without an obvious inflection point. Higher BMI was associated with a higher probability of HUA. In contrast, the association between WC and HUA was more complex, showing an increase followed by a decrease, suggesting non-linear relationships between WC and HUA.

### 3.5 Influence of liver enzymes on the associations between metabolic obesity phenotypes and HUA

In the BMI-based metabolic obesity phenotypes, using MHNGO as the reference group, among individuals with normal liver enzymes, the odds of HUA were significantly higher in those with MUNGO (OR = 1.60, 95% CI: 1.04–2.53; *p* < 0.05) and MUGO (OR = 4.23, 95% CI: 2.76–6.63; *p* < 0.01), whereas no significant association was observed for MHGO (Table 4). Among individuals with elevated liver enzymes, MUGO was associated with a significantly increased odds of HUA (OR = 4.77, 95% CI: 1.74–15.55; *p* < 0.01), while MUNGO and MHGO showed no significant associations.

**Table 4:** Effects of liver enzyme status on the associations between obesity phenotypes and HUA.

Obesity phenotype		Normal liver enzymes	Elevated liver enzymes
BMI-based phenotypes	MHNGO	1.00	1.00
	MUNGO	1.60 (1.04 ~ 2.53)*	1.59 (0.55 ~ 5.38)
	MHGO	1.72 (0.77 ~ 3.70)	1.92 (0.40 ~ 9.52)
	MUGO	4.23 (2.76 ~ 6.63)**	4.77 (1.74 ~ 15.55)**
WC-based phenotypes	MHNAO	1.00	1.00
	MUNAO	1.83 (1.21 ~ 2.81)**	1.87 (0.69 ~ 5.73)
	MHAO	1.73 (0.70 ~ 4.02)	1.67 (0.33 ~ 8.19)
	MUAO	4.08 (2.68 ~ 6.35)**	4.40 (1.66 ~ 13.24)**

Models were adjusted for sex, age, marital status, educational level, job type, job tenure, shift work, dietary regularity, taste preference, habit of eating sweets, smoking status, alcohol consumption, regular physical activity and nightly sleep duration. Liver enzyme categories were defined according to the diagnostic criteria for abnormal liver enzymes. MHNGO, metabolically healthy non-generalized obesity; MUNGO, metabolically unhealthy non-generalized obesity; MHGO, metabolically healthy generalized obesity; MUGO, metabolically unhealthy generalized obesity; MHNAO, metabolically healthy non-abdominal obesity; MUNAO, metabolically unhealthy non-abdominal obesity; MHAO, metabolically healthy abdominal obesity; MUAO, metabolically unhealthy abdominal obesity. *p* < 0.05*, *p* < 0.01**.

In the WC-based metabolic obesity phenotypes, using MHNAO as the reference group, among participants with normal liver enzymes, MUNAO was associated with higher odds of HUA (OR = 1.83, 95% CI: 1.21–2.81; *p* < 0.01), and MUAO showed an even stronger association (OR = 4.08, 95% CI: 2.68–6.35; *p* < 0.01), whereas no significant association was found for MHAO (Table 4). In the subgroup with elevated liver enzymes, MUAO remained significantly associated with HUA (OR = 4.40, 95% CI: 1.66–13.24; *p* < 0.01), while MUNAO and MHAO were not significantly associated. These results indicate that liver enzyme status modifies the associations between metabolic obesity phenotypes and HUA, and metabolically unhealthy obesity phenotypes are more likely to be associated with HUA.

## 4 Discussion

In this study conducted among oilfield workers, we systematically examined the associations between different obesity–metabolic phenotypes and HUA, and further explored the modifying role of liver enzymes in these relationships. Our findings indicate that obesity, particularly metabolically unhealthy obesity phenotypes (MUGO and MUAO), is significantly associated with higher odds of HUA, and that elevated liver enzyme levels further strengthen these associations. It should be noted that, given the cross-sectional design of this study, causal direction cannot be determined, and the relationships among liver dysfunction, insulin resistance, and hyperuricemia are likely bidirectional.

In our population, individuals with HUA were characterized by younger age, a higher proportion of males, lower educational attainment, and shorter job tenure. Moreover, they exhibited higher prevalence of both GO and AO, as well as less favorable metabolic profiles (e.g., BP, blood lipids) and liver enzyme levels compared with non-HUA individuals. These patterns are closely related to the specific occupational environment of oilfield workers: jobs such as oil extraction are characterized by high physical workload (27), shift work easily disrupts regular eating and sleeping patterns (28), the workforce is predominantly male (29, 30), and lifestyle factors shaped by traditional occupational culture (e.g., social drinking, insufficient physical activity) further increase the risks of obesity and metabolic disorders, thereby predisposing this group to a higher burden of HUA.

We found that both GO and AO were significantly correlated with higher odds of HUA. This is consistent with previous evidence that obesity can promote purine synthesis, reduce renal uric acid excretion, and induce insulin resistance, ultimately leading to elevated SUA levels (31). In addition, our results showed a sustained positive association between BMI and HUA, whereas the association between WC and HUA appeared nonlinear. This non-linear pattern may reflect several factors: a “tail effect” due to a smaller number of individuals with extremely high WC, potential behavioral or clinical interventions such as urate-lowering therapy in the most obese participants, or physiological phenomena sometimes referred to as the “obesity paradox,” in which very high abdominal adiposity does not linearly increase risk due to adaptive metabolic or pharmacological effects (32). This discrepancy likely reflects the distinct biological meanings of these anthropometric indicators (33): BMI primarily reflects overall adiposity and is more directly and linearly related to total metabolic load; WC reflects abdominal adiposity, and its relationship with HUA is influenced by visceral fat distribution, local inflammatory cytokine release, and other complex factors, with potential inter-individual variability (34). These findings suggest that, when assessing obesity-related HUA risk in occupational populations, it is important to integrate multiple anthropometric indices such as BMI and WC to more accurately identify high-risk individuals.

A substantial body of literature has confirmed that individuals with obesity may present with heterogeneous metabolic phenotypes (35–37). In our analysis based on obesity phenotypes defined jointly by BMI/WC and metabolic health status, we observed that metabolically unhealthy obese phenotypes were strongly associated with an elevated risk of HUA, whereas metabolically healthy obese phenotypes showed associations only in some models or even no significant increase in risk. These findings support the pathological heterogeneity between MHO and metabolically unhealthy obesity phenotypes (MUGO and MUAO): obesity per se is not the direct determinant of HUA; rather, metabolic dysregulation constitutes the core driving mechanism (38). Metabolic derangements may promote the development of HUA via multiple pathways, such as insulin resistance–induced reductions in renal tubular uric acid excretion (39), dyslipidemia–mediated increases in hepatic purine metabolic load (40), and chronic inflammation–driven enhancement of uric acid production (41). Overall, metabolic status is a key modifier of obesity-related disease risk.

Of particular note, we observed a pronounced association between abnormal liver enzymes and metabolically unhealthy obesity phenotypes (MUGO and MUAO) in relation to HUA, rather than implying direct causation. Among individuals with elevated liver enzyme levels (defined as having at least one of ALT, AST, ALP, or GGT above the reference range, to capture overall hepatic dysfunction while avoiding sparse subgroup data), the risks of HUA in those with MUGO and MUAO were further increased. A plausible mechanism is that overweight and obesity contribute to fatty liver and hepatic steatosis, accompanied by oxidative stress and inflammation in adipose tissue. As the central organ of uric acid metabolism (42), the steatotic liver is prone to hepatocellular injury, with elevated serum levels of ALT, AST and GGT representing potential manifestations of hepatic metabolic stress (43–45). Hepatocellular injury may contribute to insulin resistance, and conversely, HUA itself can induce oxidative stress and inflammatory responses that further impair liver function, suggesting a bidirectional interaction rather than a strictly unidirectional causal pathway (46, 47). Thus, the observed “vicious cycle” likely reflects complex, reciprocal interactions between liver dysfunction, insulin resistance, and hyperuricemia, rather than a simple linear sequence of events. Liver-injury–induced insulin resistance may exert a dual impact on uric acid metabolism: on the one hand, it reduces renal uric acid excretion (48), leading to increased SUA levels, consistent with the findings of Molla and colleagues (26, 49); on the other hand, it activates the hexose monophosphate shunt (pentose phosphate pathway) (50), thereby promoting purine synthesis and further increasing SUA levels, which in turn aggravates hepatic fat accumulation (51). Simultaneously, insulin resistance disrupts glucose and lipid metabolism, exacerbating global metabolic disturbances (52). Against this background of metabolic dysregulation, HUA can further progress; in addition, HUA itself induces oxidative stress in adipose tissue and triggers adipose inflammation, thereby aggravating insulin resistance (53). Persistent metabolic disturbances continuously impair liver function and worsen hepatic injury, which is difficult to reverse due to the ongoing presence of obesity and metabolic abnormalities. Ultimately, this may lead to a vicious cycle in which “obesity aggravates hepatic injury, hepatic injury promotes insulin resistance, insulin resistance worsens metabolic dysfunction, and metabolic dysfunction is associated with HUA while reinforcing each upstream step.” These results suggest that abnormal liver enzymes may constitute a key pathological hub linking MUGO, MUAO and HUA.

The present findings have important implications for metabolic health management in oilfield workers. First, routine monitoring of liver function should be incorporated into occupational health examinations to facilitate early identification of individuals at elevated metabolic risk. Second, for workers with metabolically unhealthy obesity phenotypes (MUGO and MUAO), interventions should not be limited to weight control but should also target metabolic improvement and liver function, including optimization of dietary patterns, reduction of night-shift exposure and promotion of regular physical activity, in order to reduce the risk of HUA and related metabolic diseases. Finally, future occupational health strategies should aim to develop comprehensive risk assessment models that integrate body weight, metabolic status and liver function indices, thereby providing a more accurate basis for precision prevention in this population.

Several limitations of this study should be acknowledged. First, the cross-sectional design precludes causal inference regarding the relationships among obesity–metabolic phenotypes, liver enzymes, and HUA, and reverse causality cannot be excluded. Second, although a proportion of participants were excluded because of missing anthropometric data, supplementary analyses indicated that the included participants were broadly representative of the eligible population; nevertheless, some degree of residual selection bias cannot be ruled out. Third, lifestyle factors were assessed in a relatively crude manner. In particular, alcohol consumption was collected only as a binary variable, without quantification of intake volume or differentiation by beverage type (e.g., beer vs. spirits). Similarly, other lifestyle factors, including diet composition and physical activity intensity, were not quantitatively assessed. Given that hyperuricemia is strongly influenced by purine-rich foods, fructose intake, and the quantity and type of alcohol consumed, these limitations may affect the interpretation of the observed associations between liver enzymes, metabolic status, and HUA. In addition, information on the use of urate-lowering therapy or medications that may influence liver enzyme levels was unavailable, which could have resulted in residual confounding. Finally, as the study population consisted of actively employed oilfield workers from a single enterprise, a healthy worker effect may exist, potentially leading to an underestimation of the prevalence of HUA and related metabolic abnormalities and limiting the generalizability of the findings. Future prospective, multi-center studies with more detailed exposure assessment and comprehensive lifestyle characterization are warranted.

## 5 Conclusion

In this occupational population, obesity—particularly metabolically unhealthy general and abdominal obesity—was significantly associated with an increased risk of hyperuricemia, while elevated liver enzyme levels were independently related to HUA. BMI showed a linear association with HUA, whereas waist circumference exhibited a nonlinear relationship, suggesting distinct roles of general and abdominal adiposity. These findings underscore the importance of hepatic metabolic dysfunction in the obesity–HUA link. Integrated monitoring of adiposity, metabolic phenotypes, and liver function may facilitate early identification and prevention of HUA in high-risk working populations.

## Data Availability

The raw data supporting the conclusions of this article will be made available by the authors, without undue reservation.
