# Supplementary material for: Association between different metabolic obesity phenotypes and hyperuricemia: the modifying role of liver enzymes
Source: Front Public Health. 2026 Feb 20;14:1764219. doi: 10.3389/fpubh.2026.1764219 (PMC12962922; doi:10.3389/fpubh.2026.1764219)
Supplement: Supplementary file 1 [file Data_Sheet_1.pdf]

Supplementary Table S1 Baseline characteristics of included participants compared with the total eligible population.

**Variables**		**included participants n=1867**	**overall eligible population n = 3107**	***p* value**
Sex, n/%	Male	1235 (66.1)	1991 (64.1)	0.139
	Female	632 (33.9)	1116 (35.9)	
Age (years), n/%	23-35	464 (24.9)	845 (27.2)	0.191
	36-50	1153 (61.8)	1861 (59.9)	
	51-61	250 (13.4)	401 (12.9)	
Marital status, n/%	Married	1561 (83.6)	2593 (83.5)	0.888
	Other (single, divorced, widowed)	306 (16.4)	514 (16.5)	
Educational level, n/%	Bachelor's degree or above	542 (29.0)	948 (30.5)	0.542
	Junior college	529 (28.3)	865 (27.8)	
	High school or below	796 (42.6)	1294 (41.6)	
Job type, n/%	Oil extraction worker	1245 (66.7)	1816 (58.4)	<0.001
	Other	622 (33.3)	1291 (41.6)	
Job tenure, n/%	≤13	539 (28.9)	986 (31.7)	0.154
	14-17	425 (22.8)	705 (22.7)	
	18-27	520 (27.9)	803 (25.8)	
	≥27	383 (20.5)	613 (19.7)	
Shift work status, n/%	None	289 (15.5)	547 (17.6)	0.058
	Yes, without night shifts	456 (24.4)	792 (25.5)	
	Yes, with night shifts	1122 (60.1)	1768 (56.9)	
Eating pattern, n/%	Regular	1502 (80.4)	2359 (81.7)	0.299
	Irregular	365 (19.6)	530 (18.3)	

Variables		included participants n=1867	overall eligible population n = 3107	*p* value
Taste preference, n/%	Light	329 (17.6)	507 (17.5)	0.939
	Moderate	1279 (68.5)	1991 (68.9)	
	Salty	259 (13.9)	391 (13.5)	
Habit of eating sweets, n/%	No	1366 (73.2)	2100 (72.7)	0.718
	Yes	501 (26.8)	789 (27.3)	
Smoking status, n/%	Non-smoker	1172 (62.8)	1848 (64.0)	0.404
	Smoker	695 (37.2)	1041 (36.0)	
Alcohol consumption, n/%	Non-drinker	1450 (77.7)	2245 (77.7)	0.972
	Drinker	417 (22.3)	644 (22.3)	
Physical activity, n/%	Regular	668 (35.8)	1060 (36.7)	0.813
	Occasional	1051 (56.3)	1605 (55.6)	
	Never	148 (7.9)	224 (7.8)	
Nightly sleep duration (hours), n/%	7-8	634 (34.0)	992 (34.3)	0.682
	5-6	1025 (54.9)	1555 (53.8)	
	<5	208 (11.1)	342 (11.8)	
GO, n/%	Normal weight	1001 (53.6)	1677 (55.3)	0.258
	Obese	866 (46.4)	1357 (44.7)	
AO, n/%	Normal	1125 (60.3)	1320 (61.2)	0.555
	AO	742 (39.7)	838 (38.8)	
Age		41.6±8.0	41.3±8.2	0.231
BMI		24.9±4.0	24.9±4.0	0.744

**Variables**	**included participants n=1867**	**overall eligible population n = 3107**	***p* value**
WC	84.4±11.7	84.3±11.7	0.959
SBP	115 (105, 127)	115 (104, 126)	0.104
DBP	76 (68, 84)	75 (68, 83)	0.165
TG	1.6 (1.1, 2.4)	1.5 (1.0, 2.4)	0.035
HDL-C	1.04 (0.89, 1.22)	1.04 (0.88, 1.22)	0.358
FBG	4.8 (4.5, 5.2)	4.8 (4.5, 5.2)	0.196
ALT	21 (14, 32)	20 (13, 31)	0.138
AST	19 (16, 23)	18 (16, 23)	0.056
ALP	73 (60, 89)	72 (59, 88)	0.093
GGT	25 (16, 40)	24 (15, 38)	0.239
